# Autophagy: A necessary process during the *Trypanosoma cruzi* life-cycle

**DOI:** 10.1080/21505594.2018.1543517

**Published:** 2018-11-29

**Authors:** Betiana Nebaí Salassa, Patricia Silvia Romano

**Affiliations:** a Laboratorio de Biología de Trypanosoma cruzi y la célula hospedadora, Instituto de Histología y Embriología (IHEM) “Dr. Mario H. Burgos” CONICET, Universidad Nacional de Cuyo, Mendoza, Argentina; b Facultad de Odontología, Universidad Nacional de Cuyo, Mendoza, Argentina; c Facultad de Ciencias Médicas, Universidad Nacional de Cuyo, Mendoza, Argentina

**Keywords:** *T. cruzi*, metacyclogenesis, Atg8.1, *T. cruzi* infection, host cell, LC3

## Abstract

Autophagy is a well-conserved process of self-digestion of intracellular components. *T. cruzi* is a protozoan parasite with a complex life-cycle that involves insect vectors and mammalian hosts. Like other eukaryotic organisms, *T. cruzi* possesses an autophagic pathway that is activated during metacyclogenesis, the process that generates the infective forms of parasites. In addition, it has been demonstrated that mammalian autophagy has a role during host cell invasion by *T. cruzi*, and that *T. cruzi* can modulate this process to its own benefit. This review describes the latest findings concerning the participation of autophagy in both the *T. cruzi* differentiation processes and during the interaction of parasites within the host cells. Data to date suggest parasite autophagy is important for parasite survival and differentiation, which offers interesting prospects for therapeutic strategies. Additionally, the interruption of mammalian autophagy reduces the parasite infectivity, interfering with the intracellular cycle of *T. cruzi* inside the host. However, the impact on other stages of development, such as the intracellular replication of parasites is still not clearly understood. Further studies in this matter are necessaries to define the integral effect of autophagy on *T. cruzi* infection with both *in vitro* and *in vivo* approaches.

## Introduction

Successful parasites have evolved to live and develop in specific hosts during their biological cycle. They have accumulated adaptations in response to environmental changes such as the immune response of the host. The repetition of the parasite-host interaction cycle ensures the persistence of several species of parasites. In humans, this interaction causes health disorders known as vector-borne and parasitic diseases.

According to the WHO, human parasitic diseases cause more than 700,000 deaths each year (http://www.who.int/vector-control/en/). These infectious diseases are caused by parasites from the group of protozoa and helminths. Some species of arthropods are also parasites and produce the so-called ectoparasitic diseases. Many of these diseases are classified as Neglected Tropical Diseases (NTDs) because they are caused by pathogens present in tropical and sub-tropical areas, and are often overlooked by pharmaceutical companies and even the public health community.

Trypanosomatidae is a vast family of protozoan parasites characterized by the presence of a single flagellum and a compacted DNA called kinetoplast. All members are exclusively parasitic, found primarily in insects. A few genera have life-cycles involving a secondary host, which may be a vertebrate, invertebrate or plant. These include several species that cause major diseases in humans [1]. The three major human diseases caused by trypanosomatids are Leishmaniasis, caused by various species of the genus Leishmania; African trypanosomiasis (or “sleeping sickness”), caused by *Trypanosoma brucei*; and American trypanosomiasis or Chagas disease, caused by *Trypanosoma cruzi*. These digenetic parasites have complex biological cycles in response to the specific adaptations required to live within two very different types of hosts, the insect vector and the mammalian organism.

Autophagy is an intracellular catabolic pathway highly conserved in eukaryotic organisms. Experimental and genomic data showed that the autophagy machinery characterized in animals and fungi also appeared in protists and function for diverse lifestyle adaptations [2]. Pathogenic protists can either utilize their own autophagy mechanisms or manipulate host-cell autophagy in order to establish or maintain infection within a host. This work revises the involvement of *T. cruzi* and host cell autophagy during the *T. cruzi* life-cycle. 

## The *T. cruzi* life-cycle and the infection process

During its complex life cycle, *T. cruzi* is found in three different parasitic forms. Epimastigotes and amastigotes are the replicative stages found in the lumen of the intestine of triatomine insect vectors or in the cytosol of infected host cells, respectively. The infective forms of *T. cruzi* are metacyclic trypomastigotes and blood-stream trypomastigotes, derived from epimastigotes and amastigotes, respectively, both with the capacity to invade a large number of different cell types in a process independent of host cell actin polymerization [3]. The so-called extracellular amastigotes, originated from the premature rupture of infected cells or transformed from swimming trypomastigotes, are also infective forms that, in contrast to trypomastigotes, require functional intact microfilaments to infect non-phagocytic host cells [4,5].

**Figure 1.**
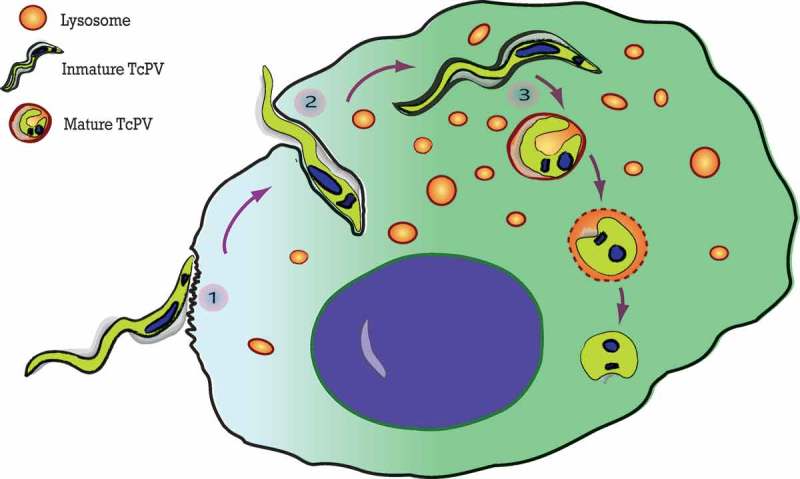
The three steps of the *T. cruzi* invasion. The scheme summarizes the process of *T. cruzi* entry into the host cell. (1) Adhesion of trypomastigotes to the host cell surface. (2) Internalization of trypomastigotes produced by invagination of the plasma membrane. (3) Vacuole maturation proceeds after the fusion with lysosomes which initiates the differentiation of *T. cruzi* from trypomastigotes to amastigotes.

The *T. cruzi* entry process was classically divided into two specific steps (Figure 1, points 1 and 2): the adhesion process, related to the binding of *T. cruzi* antigens to host cell receptors which trigger cell signaling events [6,7]; and the internalization process that describes the mechanism that culminates in the formation of the *T. cruzi* parasitophorous vacuole (TcPV). Adhesion of trypomastigotes is driven by a repertoire of molecules present at the parasite surface or secreted during the infection process which bind to the specific receptors in the host cells [6,8,9]. Despite the advances made towards identification of the receptor molecule responsible for the adhesion of parasites, no single main host receptor candidate has been identified yet. In fact, the arsenal of parasite surface proteins and the multiple possible host receptors provide several opportunities for *T. cruzi* to recognize and contact cells and explains why this parasite is able to infect almost all types of mammalian cells [10,11]. The adhesion to cell surface triggers a host cell signaling cascade that culminates in the formation of the *T. cruzi* parasitophorous vacuole. Different processes have been described for the internalization of *T. cruzi* into non-phagocytic cells. Seminal studies showed two different models of trypomastigote invasion: by activating the lysosomal exocytosis or by inducing an endocytosis-like process. Both agree on the absence of plasma membrane protrusions (and microfilaments) but differ in the source of the membrane that initially envelops the parasite. The first model demonstrated that parasites elicited a Ca2+ signaling cascade in the host cells which activates lysosomal exocytosis [12]. The later fusion of lysosomes with the host plasma membrane allows the parasite entry and the formation of a parasitophorous vacuole with lysosomal characteristics [13]. This lysosomal exocytosis is produced with the peripheral pool of lysosomes and require microtubules and kinesin to the lysosomal transport toward the plasma membrane [14,15]. Autophagy has also been shown to participate in this process by the finding of autophagic proteins in the TcPV membrane [16] (see below). In contrast, the endocytosis-like process claims that at very early times after infection, the highest proportion of trypomastigotes enter host cells by invagination of the plasma membrane that generates an initial TcPV enriched in phosphoinositides derived from plasma membrane but not lysosomal markers [17]. This model reinforced previously published data that demonstrated the participation of endocytic GTPases such as Rab5 and Rab7 and dynamin in the *T. cruzi* infection process [18,19]. Later studies showed that *T. cruzi* exploited the plasma membrane wound repair mechanism [20] carried out by the exocytosis of lysosomes at the site of the injury followed by the compensatory endocytosis of injured membranes [21]. In summary, both events, exocytosis and endocytosis, previously classified as independent processes, now, under the light of these new data, have been shown to occur sequentially during parasite invasion. The different styles of *T. cruzi* entry were also associated with the different trypomastigote forms. Metacyclic trypomastigotes (MT) that express gp82, the surface molecule that mediates invasion, induce mTOR dephosphorylation and lysosome biogenesis and scattering, evidencing preferentially the lysosomal exocytosis process. In contrast, the entry of tissue culture trypomastigotes (TCT), the equivalent to blood-stream trypomastigotes, is predominantly related to endocytosis [22]. Other forms of *T. cruzi* entry into host cells, such as phagocytosis and macropinocytosis, have been also described [23].

In addition to the specific manner of TcPV formation, there is a third little described step during *T. cruzi* invasion that involves the TcPV maturation (Figure 1, point 3). Fusion of lysosomes with the early parasitophorous vacuole is crucial to the progress of infection. Previous works from the laboratory of Norma Andrews have demonstrated that when lysosomal fusion was inhibited, internalized parasites failed to be retained inside host cells and escaped to the extracellular environment [24,25]. The abolition of this phenomenon, known as reversal infection, by allowing the lysosomal fusion to TcPV, is a key process for the retention of *T. cruzi* inside the host cell [26]. In agreement with this, further studies from our group demonstrated that the SNARE VAMP7 and its partner Vti1b, both components of the molecular machinery that promotes lysosomal fusion, are required during *T. cruzi* vacuole development and, as a consequence, for the parasite infection [27]. We have also shown that migration of VAMP7 positive vesicles to the TcPV depends on KIF5, a motor protein belonging to the superfamily of kinesins. It was suggested that the highly motile trypomastigotes contained in vacuoles fail to associate with microtubules when lysosomal fusion is impaired, allowing the parasites to continue to move around the cytosol and eventually leave the cell. In the same way, the loss of parasite motility associated with the lysosomal fusion to the TcPV could be attributed to the beginning of the differentiation process from trypomastigotes to non-motile amastigotes induced in part by the acidic pH gained by the vacuole during this process. The striking morphological transition undergone by the parasite throughout the course of the infection process, from the typical elongated form at early times to the ovoid form at later times [27] strengthens this idea. Maturation of the vacuole is finally a key process for both the retention of *T. cruzi* inside the host cell and for the progression of the *T. cruzi* intracellular cycle by allowing the differentiation from trypomastigotes to amastigotes.

## The autophagic pathway

Autophagy is a catabolic pathway conserved in most eukaryotes from yeast to mammalian cells [28]. This process involves the cellular self-digestion through the lysosomal machinery. Macroautophagy, the most widely studied type of autophagy, is important in many physiological situations such as cell development, cell growth, and cell differentiation. As a constitutive process, autophagy functions at basal levels in the turnover of long-lived proteins and old organelles for maintaining cellular homeostasis. Nutritional stress conditions are the main physiologic stimulus of autophagy. Under these situations, cells entrap cytoplasmic materials and degrade them to provide aminoacids that function as energy source for cell survival [29]. Besides this bulk degradation process, autophagy functions selectively to remove misfolded or aggregated proteins, damaged organelles, such as mitochondria, endoplasmic reticulum and peroxisomes, as well as to eliminate intracellular pathogens, being part of the innate immune response [30]. The autophagic pathway involves specific compartments inside the cell. Basically, the cytoplasmic cargo is captured through the intermediary of a double membrane-bound vesicle, referred to as autophagosome, which fuses with the lysosome to form an autolysosome. In mammalian cells, autophagosomes derived from an isolation membrane, also known as phagophore, which is likely derived from a lipid bilayer contributed by the endoplasmic reticulum and/or the trans-Golgi and late endosomes [31–33]. The outer mitochondrial membrane and the plasma membrane can also participate as membrane donor [34]; therefore, the exact origin of the phagophore in mammalian cells is still controversial. The isolation membrane grows around the cargo and finally closes to form the early autophagosome, easily recognized as a double-membrane vesicle by electron microscopy [35]. Autophagosomes can then fuse with endocytic compartments to form amphisomes, which, in turn, fuse with lysosomes, forming autolysosomes where the proteins are degraded to amino acids that are recycled via lysosomal permeases and other transporters [36].

Specific molecules coordinate this complex process. To date, different autophagy-related genes (Atg) have been identified by genetic screening in yeast [28]. Many of them have been conserved in mammalian cells. These genes can be grouped according to their functions at key stages of the autophagy pathway: initiation, elongation, maturation, and fusion with the lysosomes. In mammalian systems, the phagophore is derived from membranes enriched in phosphatidyl inositol triphosphate (PI3P), the product of the Beclin1-Vps34 complex [37]. Phagophore elongation requires the sequential activation of two protein conjugation reactions. The mammalian Atg5-Atg12-Atg16L complex is recruited to the isolation membrane, favoring the extension of the precursor membrane [38]. LC3, the mammalian homolog of Atg8, is then conjugated with phosphatidylethanolamine (PE) to form LC3-II, which inserts into the autophagosome membrane and contributes to vesicle elongation [39]. After autophagic induction LC3-II is visualized by a punctuate pattern corresponding to autophagic vesicles. Unlike the Atg5-Atg12.Atg16L complex, LC3-II remains on completed autophagosomes and also on autolysosomes [40]. The final steps of autophagy involve docking and fusion of autophagosomes to lysosomes to generate autolysosomes which produce cargo degradation and recycling. Specific Rab GTPases and SNAREs proteins function as master regulators of these fusion events. Impairment of this process by the use of mutant proteins or chemicals prevents the normal maturation of the pathway leading to the accumulation of autophagosomes. Bafilomycin A1 is a broadly used autophagic flux inhibitor that prevents the fusion of autophagosomes with endosomes/lysosomes and the autophagic degradation [41].

Two key signaling processes converge to correlate canonical autophagy with cell nutrient or stress conditions, the mTOR and the PI3K pathways. The mTOR signaling cascade transduces the response from growth factors, via class I PI3K, Akt/PKB, and so forth, to negatively regulate autophagy [42]. Rapamycin is one of the classical inducers of autophagy due to inhibition of the mTOR signaling pathway [43]. As mentioned above, Beclin1-Vps34 complex is necessary to produce the PI3P required in the phagophore formation and elongation. This class III PI3K complex is inhibited by wortmannin, which blocks the autophagosome initiation process [44].

The process of autophagy can be monitored intracellularly by utilizing LC3 fused to a fluorescent protein such as GFP-LC3 or mCherry-LC3. Fluorescent LC3 is incorporated into the autophagosomes and could be observed as small dots inside the cell by light microscopy. The number of puncta in a cell at any specific time is a function of both the formation and the clearance of autophagosomes [45].

Selective autophagy refers to the different mechanisms of capture and degradation of specific cargos by autophagy. These non-canonical classes of autophagy are named according to the type of component that has been incorporated to the route, such as mitophagy, pexophagy and xenophagy [46], among others. Xenophagy is the process by which cells can degrade an intracellular microorganism present inside a phagosome [47,48] or free in the cytosol [49]. Due to this property, xenophagy has been classified as a component of the innate immune responses against intracellular pathogens. Xenophagy generally functions as a second barrier when phagocytosis or other defense mechanisms are exceeded. However, some pathogens have the capacity to evade autophagic responses or to subvert the autophagic pathway and to live and replicate inside an autophagosomal compartment (see below). Recent research has related LC3 protein to pathways other than autophagy. Since 2007, the concept of LAP (LC3-associated phagocytosis) has been introduced. Nowadays LAP is understood as a type of non-canonical autophagy. LAP uses some autophagic components but it differs mainly in that the LC3 positive vesicle is a simple membrane compartment [50,51].

## Autophagy during the *T. cruzi* life-cycle

As eukaryotic organisms, protists possess a rudimentary autophagic pathway that functions at specific stages of their life-cycles mainly during the differentiation processes [2]. Autophagy is also activated when pathogenic protists are treated with anti-parasitic drugs, as a mechanism to overcome the stress caused by the toxic compounds before cell death [52–55]. In addition, a growing body of evidence shows that intracellular protists have the ability to manipulate host-cell autophagy in order to establish or maintain the infection within a host. Below we summarize the findings made on the *T. cruzi* autophagy pathway and its participation in the differentiation processes, and then we describe the recent advances concerning the interaction of *T. cruzi* with host cell autophagy.

## Autophagy is induced during the *T. cruzi* differentiation processes

The first evidence for the existence of an autophagic-like process in *T. cruzi* was provided, as in mammalian cells, by morphological studies showing the presence of double-membrane vesicles and multivesicular structures in parasites treated with trypanocidal drugs [56,57]. Later genome database searches supplemented with more advanced analyses revealed the presence of autophagy-associated components in *T. cruzi* [58–60]. These bioinformatics analyses showed that only half of the genes found in yeasts were present in trypanosomatids. *T. cruzi* possesses all components of the LC3/Atg8 conjugation system, but lacks the Atg5-Atg12 protein complex, indicative of a different manner to initiate and elongate the autophagosome. Two TcAtg8 homologs have been found in *T. cruzi*: TcAtg8.1 and TcAtg8.2. Only the first one was functionally linked to autophagy [60]. During starvation, TcAtg8.1 is efficiently cleaved by one of the two TcAtg4 homologs (autophagins) and then, after conjugation to PE, is inserted in the membrane of the autophagosome-like vesicles [60]. Further studies from our lab established the participation of autophagy during the differentiation of *T. cruzi* from epimastigotes to metacyclic trypomastigotes [61]. This process, called metacyclogenesis, takes place in the lumen of the intestine of the triatomine vector. The reduced nutrient availability produced by the high multiplication of epimastigotes is the main stimulus to induce it. By performing an *in vitro* method of differentiation, we showed an increment in the number of Atg8.1 positive vesicles during starvation. The same result was observed in the presence of other autophagy inducers such as rapamycin, the classical inhibitor of TOR kinase, and spermidine, a polyamine that induces autophagy in many eukaryotic organisms by regulation of gene expression and protein activity [62]. We also showed that classical autophagy inhibitors such as wortmannin impaired the autophagosome formation in *T. cruzi* [61]. In agreement with this, Schoijet et al. [63] showed that TcVps34 kinase (the parasitic counterpart of the mammalian Beclin-1-Vps34 kinase) is regulated by TcVps15 and that both form a complex that participates in starvation induced autophagy in *T. cruzi*. As mentioned above, bafilomycin is a H^+^ pump inhibitor that impairs the normal autophagic flux, producing an accumulation of autophagic structures [41]. In contrast to mammalian cells, in *T. cruzi* bafilomycin inhibits the first steps of autophagy, evidenced by the low number of Atg8.1 positive vesicles generated under this treatment. A previous work showed that formation of autophagosome in the related parasite *T. brucei* requires the normal acidification of acidocalcisomes, acidic parasitic compartments required for osmoregulation [64]. Acidification impairment of acidocalcisomes by bafilomycin treatment completely abrogates autophagic response, indicating that these compartments could be participating in the first steps of autophagy in trypanosomes in a not yet clearly understood mechanism.

Autophagy is also required during the differentiation of trypomastigotes to amastigotes, a process that starts in the acidic environment of the parasitophorous vacuole and finish in the host cell cytosol. Our previous data showed that starvation in PBS and rapamycin were able to induce this process similarly to low pH. Moreover, we observed Atg8-decorated vesicles in amastigotes present in the cytosol of infected cells, indicating that autophagy can be induced in the parasite inside the host [16]. Although more studies will be necessary in the future to elucidate the specific mechanisms that regulate autophagy in trypanosomatids, these data show that this process is an interesting target to be modulated to impair *T. cruzi* survival in the host.

## Host autophagy during *T. cruzi* infection

### Invasion of trypomastigotes

**Figure 2.**
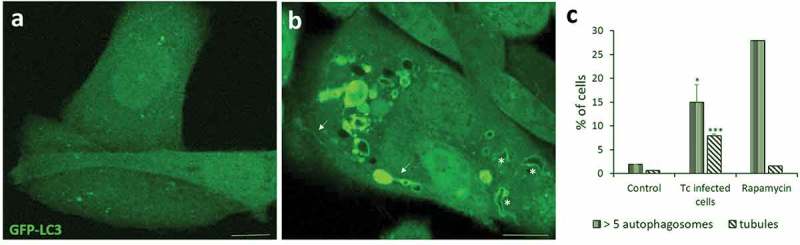
Autophagy is induced during *T. cruzi* infection. CHO cells overexpressing GFP-LC3 were infected with tissue culture trypomastigotes (TCT) of *T. cruzi* Y strain (MOI = 50) during 3 h. After fixation, cells were washed, mounted in mowiol and analyzed by confocal microscopy. (a) Control (non-infected) cells depicting the homogeneous distribution of GFP-LC3 in the soluble form. (b) Infected cell displaying numerous autophagic vacuoles and tubules (arrows) decorated with membrane-associated GFP-LC3. Note the recruitment of this protein in the membrane of the *T. cruzi* parasitophorous vacuole (asterisks) that were recognized by the typical form of trypomastigotes. (c) Percentage of cells containing more than 5 LC3-positive vesicles/cell and at least one tubule/cell at the indicated conditions. Rapamycin (50 ng/μl) were incubated for 2 h in control conditions. Scale bar: 5 μm. Data shown represent the mean ± SE from 3 independent experiments. *p < 0.05, ***p < 0.001 (Student´s t-test).

As mentioned above, the interaction between host autophagy and intracellular pathogens has different fates according to the type of microorganism and host cell. Many microorganisms can be engulfed by autophagic compartments and eliminated in an autolysosome by xenophagy. Another group of pathogens has the ability to subvert the autophagic pathway to its own benefit. Some pathogens inhibit the autophagic flux to replicate in an autophagic niche which does not fuse with lysosomes [65]. Others have evolved to live and replicate inside a compartment with autolysosomal characteristics [66–69]. In the case of *T. cruzi*, our data showed that this parasite exploits autophagy to support invasionof the host cell. Previous studies from our laboratory demonstrated that parasite entry by the lysosomal route is favored under conditions that increase autophagy [16,70]. Induction of autophagy before infection by starvation or other means significantly increased the percentage of infected cells by enhancing the number of lysosomal compartments (lysosomes and autolysosomes) available for *T. cruzi* infection, resulting in an increased colonization of the host cell. We also showed that the *T. cruzi* parasitophorous vacuole is decorated with LC3 protein (Figure 2) and that the autophagic inhibitors wortmannin, 3-methyladenine or vinblastine suppress this recruitment and also significantly reduce the intracellular infection. Interestingly, infection was diminished in the absence of specific autophagy genes Beclin1 or Atg5, which are required for initiation of autophagy, indicating that autophagic-derived compartments are required for the increased entry of *T. cruzi* into the host cell. The pro-pathogen effects of autophagy on *T. cruzi* infection were observed in different types of host cells and of *T. cruzi* strains, demonstrating that this interaction is a widespread phenomenon [71]. These data were obtained with the TCT forms of *T. cruzi*. Quite different features were described by the group of Nobuko Yoshida, working with metacyclic trypomastigotes. They showed that, in contrast to TCT, pre-starvation of cells by 2 h or rapamycin treatment reduced MT invasion whereas other conditions that promote lysosomal exocytosis increased it [72]. Further data from the same group demonstrated that conditions that promote lysosome biogenesis and scattering such us 1 h starvation or treatment with sucrose, increase cell susceptibility to MT and resistance to TCT [22]. Due to cellular distribution of lysosomes have different outcomes related to the treatment used (1 h or 2 h starvation or rapamycin), these data indicate that MT invasion is mainly related to lysosomal exocytosis, whereas TCT entry is predominantly an endocytosis–like process.

Concerning the mechanism of *T. cruzi* invasion, it is important to note that, as mentioned above, a growing body of evidence showed that trypomastigotes exploit the lysosome-dependent membrane repair mechanism to enter the host cell [21]. In this sense, autophagy has been shown to play a role in the fixing of damaged pathogen containing vacuoles such as *Salmonella* [73]. The rupture of the cell surface produced by *T. cruzi* during invasion may trigger the autophagic response as a mechanism to restore the plasma membrane integrity. In agreement with this hypothesis, our results using live imaging by confocal microscopy showed that GFP-LC3 positive vesicles move towards the plasma membrane and contact the sites where trypomastigotes bind to the membrane [16,71].

A recent work showed that autophagosome formation was induced by *T. cruzi* infection. By quantifying the number of LC3 puncta in cells infected with *T. cruzi*, the authors demonstrated a gradual increment of these vesicles, which reach a maximum level at 9 h post infection [74]. Unpublished data from our laboratory show that the percentage of cells with more than five LC3-positive vesicles/cell was significantly increased in cells infected with TCT of *T. cruzi* Y strain, compared to control (Figure 2). Interestingly, we also observed a significantly higher number of LC3-positive tubules in these cells, which has not been observed either in the uninfected control cells or in rapamycin treated cells (Figure 2). Some of these tubules are even associated with the TcPV. Previous works have associated this type of dynamic tubules with efficient pathogen proliferation [75,76]. Induction of these tubules in cells infected with *T. cruzi* could favor the arrival or elimination of specific molecules into the TcPV, creating a favorable niche for the parasite. More experiments will need to confirm this hypothesis.

Onisuka and colleagues [74] also showed that *T. cruzi* infection inhibited the normal maturation of autophagosomes to autolysosomes (autophagic flux). Our investigation concerning this point was not conclusive. By using the GFP-RFP-LC3 tandem, we did not observe a different pattern of green and red colocalization, compared to control. Western blot experiments also yielded the same ambiguous results (data not shown). Unexpectedly, colocalization between DQ-BSA, one of the best markers of hydrolytic compartments, with the *T. cruzi* vacuole at different times after infection was lower compared to the marker of acidic compartments lysosensor (data not shown). On the other hand, many authors, including our laboratory, found the classical late endosomal/lysosomal markers, such as VAMP7, Lamp 1 and 2 and Cathepsin D, in the TcPV by indirect immunofluorescence [13,16,27]. These evidences suggest that although lysosomal fusion to TcPV is produced during vacuole maturation, the final mature TcPV might not be as hydrolytic as supposed. If this is the case, lysosomal enzymes might not be fully delivered to the vacuole or remain with low activity inside de TcPV because of the sub-optimal pH. Another possibility is that lysosomal enzymes were not activated inside the TcPV by a currently unknown mechanism. This hypothesis may explain, in addition to other mechanisms, the resistance of the parasites to lysosomal degradation. More studies will be necessary to confirm this assumption.

### Intracellular differentiation and replication of amastigotes

As mentioned above, previous studies from our laboratory showed that conditions that increase autophagy favor the differentiation *in vitro* from trypomastigotes to amastigotes [16]. Atg8 positive vesicles were also visualized in amastigotes developed in the host cells [16]. Although more studies will need to confirm these findings, these evidences suggest the participation of autophagy in the *T. cruzi* intracellular differentiation. The following stage in the *T. cruzi* intracellular cycle, the proliferation of amastigotes, was not significantly impacted by the autophagic modulation in non-phagocytic host cells, compared to cells maintained in control conditions [16]. Since *T. cruzi* has its own autophagic pathway that can be modulated by the same inducers and inhibitors of host autophagy [61], other experimental approaches will be necessary to decipher the possible action of autophagy modulation on *T. cruzi* infected cells. In this context, autophagy-deficient host cell lines can be used to specifically assess the impact of host autophagy loss. In contrast, growth of amastigotes on professional phagocytes might be affected by the autophagic response of these cells as it is described in the following section.

## Autophagy on *in vivo* models of infection

To date there is not published data about the outcome of *T. cruzi* infection *in vivo* under autophagic modulation. The pathogen/autophagy relationship on models of murine infections, with the exception of a few cases, remains little understood. The effect of autophagy as an innate immune component is easier to understand, particularly with the use of knockout mice. Studies on *M. tuberculosis* murine infections showed that Atg5 deficient mice relative to autophagy-proficient littermates resulted in increased bacillary burden and excessive pulmonary inflammation evidencing the role of autophagy *in vivo* by suppressing both *M. tuberculosis* growth and damaging inflammation [77]. Furthermore, mice deficient in either IFN-γ or IFN-γ receptors are highly susceptible to *M. tuberculosis* infection [78]. It is known that IFN-γ is a key cytokine in the regulation of the immune responses [79] that explains the high susceptibility of these mice to mycobacterial infection. This cytokine is also a strong inducer of autophagy in macrophages [80]. Taking into account that macrophage activation is a key process to control *M. tuberculosis* infection, the absence of an appropriate autophagy/xenophagy response from macrophages might also explain the higher bacterial burden observed in these mice.

The main concerns with the *in vivo* models arise from the cases of pathogens for which *in vitro* studies have shown them to be favored by autophagy induction. No current evidence demonstrates that autophagy gene deletion in the host attenuates microbial disease in these cases. Therefore, the physiological significance of microbial utilization of autophagy for “promicrobial” effects remains to be established [81]. In line with these thoughts, our group observed a more aggressive infection of *T. cruzi* in an autophagy deficient mice model compared to the wild type counterpart (Casassa et al, unpublished). We also observed higher levels of infection in peritoneal cells obtained from autophagy deficient mice than cells from control animals. This means that contrary to what was observed on *in vitro* infections, deficiency of autophagy on *in vivo* models resulted in an increased infection and parasite burden. These findings highlight the role of autophagy as a component of immune responses against *T. cruzi*. Future studies on the participation of autophagy at different levels of the immunity against *T. cruzi* will allow understanding the mechanisms that prevail in the establishment of the infection, particularly in the case of the chronic stage which is characterized by *T. cruzi* persistence, tissue damage and inflammatory response.

## Concluding remarks

As one of the main processes that regulate cell survival, differentiation or death, autophagy has a major participation in the *T. cruzi* life-cycle. Evidence shows that parasitic autophagy is required during the interconversion between epimastigotes, trypomastigotes and amastigotes. These changes confer to the parasite the capacity to adapt to the following host in their life-cycle making parasite autophagy an excellent target for trypanocidal drugs.

Host autophagy has also been described as a one of the main regulators of *T. cruzi* invasion with possible effects on the maturation of the parasitophorous vacuole. Therefore, modulation of this process could favor the infection control by the cell. Furthermore, the recruitment of LC3 to the *T. cruzi* parasitophorous vacuole constitutes an interesting finding that needs to be better characterized in the future, to understand which type of process, e.g. canonical autophagy; xenophagy or even LAP are involved.

The pivotal role of autophagy in pathogenicity and virulence demonstrated in *T. cruzi* suggests that autophagy machinery is a possible good target for anti-parasitic intervention. Further studies, especially using different classes of cells, phagocytic and non-phagocytic and alsoon *in vivo* models of infection by *T. cruzi*, will be mandatory to confirm the possible beneficial effect of autophagy modulation that might offer the perspective to interfere with the infective cycle of *T. cruzi* in the host.

## References

[1] Simpson AGB, Stevens JR, Lukeš J. The evolution and diversity of kinetoplastid flagellates. Trends Parasitol. 2006;22:168–174. 16504583 10.1016/j.pt.2006.02.006

[2] Duszenko M, Ginger ML, Brennand A, et al Autophagy in protists. Autophagy. 2011;7:127–158. 20962583 10.4161/auto.7.2.13310 PMC3039767

[3] Schenkman S, Robbins ES, Nussenzweig V Attachment of Trypanosoma cruzi to mammalian cells requires parasite energy, and invasion can be independent of the target cell cytoskeleton. Infect Immun. 1991;59:645–654. 1987081 10.1128/iai.59.2.645-654.1991 PMC257806

[4] Fernandes MC, Flannery AR, Andrews N, et al Extracellular amastigotes of Trypanosoma cruzi are potent inducers of phagocytosis in mammalian cells. Cell Microbiol. 2013;15:977–991. 23241026 10.1111/cmi.12090 PMC3638054

[5] Bonfim-Melo A, Ferreira ÉR, Mortara RA Rac1/WAVE2 and Cdc42/N-WASP participation in actin-dependent host cell invasion by extracellular amastigotes of Trypanosoma cruzi. Front Microbiol. 2018;9:360. 29541069 10.3389/fmicb.2018.00360 PMC5835522

[6] Maeda FY, Cortez C, Yoshida N Cell signaling during Trypanosoma cruzi invasion. Front Immunol. 2012;3:361. 23230440 10.3389/fimmu.2012.00361 PMC3515895

[7] Yoshida N Molecular basis of mammalian cell invasion by Trypanosoma cruzi. An Acad Bras Cienc. 2006;78:87–111. 16532210 10.1590/s0001-37652006000100010

[8] Magdesian MH, Giordano R, Ulrich H, et al Infection by Trypanosoma cruzi. Identification of a parasite ligand and its host cell receptor. J Biol Chem. 2001;276:19382–19389. 11278913 10.1074/jbc.M011474200

[9] Martins NO, de Souza RT, Cordero EM, et al Molecular characterization of a novel family of Trypanosoma cruzi Surface Membrane Proteins (TcSMP) involved in mammalian host cell invasion. PLoS Negl Trop Dis. 2015;9:e0004216. 26565791 10.1371/journal.pntd.0004216 PMC4643927

[10] de Souza W, de Carvalho TMU, Barrias ES Review on Trypanosoma cruzi: host cell interaction. Int J Cell Biol. 2010;2010:1–18. 10.1155/2010/295394 PMC2926652 20811486

[11] de Araujo-Jorge TC, Barbosa HS, Meirelles MNL, et al Trypanosoma cruzi recognition by macrophages and muscle cells: perspectives after a 15-year study. Mem Inst Oswaldo Cruz. 1992;87:43–56. 10.1590/s0074-02761992000900006 1342716

[12] Tardieux I, Nathanson MH, Andrews NW Role in host cell invasion of Trypanosoma cruzi-induced cytosolic-free Ca2+ transients. J Exp Med. 1994;179:1017–1022. 8113670 10.1084/jem.179.3.1017 PMC2191425

[13] Tardieux I, Webster P, Ravesloot J, et al Lysosome recruitment and fusion are early events required for trypanosome invasion of mammalian cells. Cell. 1992;71:1117–1130. 1473148 10.1016/s0092-8674(05)80061-3

[14] Rodríguez A, Samoff E, Rioult MG, et al Host cell invasion by trypanosomes requires lysosomes and microtubule/kinesin-mediated transport. J Cell Biol. 1996;134:349–362. 8707821 10.1083/jcb.134.2.349 PMC2120885

[15] Hissa B, de Oliveira Andrade L Trypasonoma cruzi uses a specific subset of host cell lysosomes for cell invasion. Parasitol Int. 2015;64:135–138. 25463313 10.1016/j.parint.2014.11.005

[16] Romano PS, Arboit MA, Vázquez CL, et al The autophagic pathway is a key component in the lysosomal dependent entry of Trypanosoma cruzi into the host cell. Autophagy. 2009;5:6–18. 19115481 10.4161/auto.5.1.7160

[17] Woolsey AM, Sunwoo L, Petersen CA, et al Novel PI 3-kinase-dependent mechanisms of trypanosome invasion and vacuole maturation. J Cell Sci. 2003;116:3611–3622. 12876217 10.1242/jcs.00666

[18] Wilkowsky SE, Barbieri MA, Stahl PD, et al Regulation of Trypanosoma cruzi invasion of nonphagocytic cells by the endocytically active GTPases dynamin, Rab5, and Rab7. Biochem Biophys Res Commun. 2002;291:516–521. 11855818 10.1006/bbrc.2002.6474

[19] Barrias ES, Reignault LC, De Souza W, et al Dynasore, a dynamin inhibitor, inhibits Trypanosoma cruzi entry into peritoneal macrophages. PLoS One. 2010;5:e7764. 20098746 10.1371/journal.pone.0007764 PMC2808331

[20] Tam C, Idone V, Devlin C, et al Exocytosis of acid sphingomyelinase by wounded cells promotes endocytosis and plasma membrane repair. J Cell Biol. 2010;189:1027–1038. 20530211 10.1083/jcb.201003053 PMC2886342

[21] Fernandes MC, Cortez M, Flannery AR, et al Trypanosoma cruzi subverts the sphingomyelinase-mediated plasma membrane repair pathway for cell invasion. J Exp Med. 2011;208:909–921. 21536739 10.1084/jem.20102518 PMC3092353

[22] Cortez C, Real F, Yoshida N Lysosome biogenesis/scattering increases host cell susceptibility to invasion by Trypanosoma cruzi metacyclic forms and resistance to tissue culture trypomastigotes. Cell Microbiol. 2016;18:748–760. 26572924 10.1111/cmi.12548 PMC5064668

[23] Barrias ES, de Carvalho TMU, De Souza W Trypanosoma cruzi: entry into mammalian host cells and parasitophorous vacuole formation. Front Immunol. 2013;4:186. 23914186 10.3389/fimmu.2013.00186 PMC3730053

[24] Andrade LO, Andrews NW Lysosomal fusion is essential for the retention of Trypanosoma cruzi inside host cells. J Exp Med. 2004;200:1135–1143. 15520245 10.1084/jem.20041408 PMC2211867

[25] Andrade LO, Andrews NW The Trypanosoma cruzi–host-cell interplay: location, invasion, retention. Nat Rev Microbiol. 2005;3:819–823. 16175174 10.1038/nrmicro1249

[26] Fernandes MC, Andrews NW Host cell invasion by Trypanosoma cruzi : a unique strategy that promotes persistence. FEMS Microbiol Rev. 2012;36:734–747. 22339763 10.1111/j.1574-6976.2012.00333.x PMC3319478

[27] Cueto JA, Vanrell MC, Salassa BN, et al Soluble N-ethylmaleimide-sensitive factor attachment protein receptors required during Trypanosoma cruzi parasitophorous vacuole development. Cell Microbiol. 2017;19:e12713. 10.1111/cmi.12713 27992096

[28] Nakatogawa H, Suzuki K, Kamada Y, et al Dynamics and diversity in autophagy mechanisms: lessons from yeast. Nat Rev Mol Cell Biol. 2009;10:458–467. 19491929 10.1038/nrm2708

[29] Yang Z, Huang J, Geng J, et al Atg22 recycles amino acids to link the degradative and recycling functions of autophagy. Mol Biol Cell. 2006;17:5094–5104. 17021250 10.1091/mbc.E06-06-0479 PMC1679675

[30] Glick D, Barth S, Macleod KF Autophagy: cellular and molecular mechanisms. J Pathol. 2010;221:3–12. 20225336 10.1002/path.2697 PMC2990190

[31] Axe EL, Walker SA, Manifava M, et al Autophagosome formation from membrane compartments enriched in phosphatidylinositol 3-phosphate and dynamically connected to the endoplasmic reticulum. J Cell Biol. 2008;182:685–701. 18725538 10.1083/jcb.200803137 PMC2518708

[32] Simonsen A, Tooze SA Coordination of membrane events during autophagy by multiple class III PI3-kinase complexes. J Cell Biol. 2009;186:773–782. 19797076 10.1083/jcb.200907014 PMC2753151

[33] Hayashi-Nishino M, Fujita N, Noda T, et al A subdomain of the endoplasmic reticulum forms a cradle for autophagosome formation. Nat Cell Biol. 2009;11:1433–1437. 19898463 10.1038/ncb1991

[34] Kroemer G, Mariño G, Levine B Autophagy and the integrated stress response. Mol Cell. 2010;40:280–293. 20965422 10.1016/j.molcel.2010.09.023 PMC3127250

[35] Baba M, Takeshige K, Baba N, et al Ultrastructural analysis of the autophagic process in yeast: detection of autophagosomes and their characterization. J Cell Biol. 1994;124:903–913. 8132712 10.1083/jcb.124.6.903 PMC2119983

[36] Yang Z, Klionsky DJ Permeases recycle amino acids resulting from autophagy. Autophagy. 2007;3:149–150. 17204852 10.4161/auto.3631

[37] Itakura E, Kishi C, Inoue K, et al Beclin 1 forms two distinct phosphatidylinositol 3-kinase complexes with mammalian Atg14 and UVRAG. Mol Biol Cell. 2008;19:5360–5372. 18843052 10.1091/mbc.E08-01-0080 PMC2592660

[38] Mizushima N, Kuma A, Kobayashi Y, et al Mouse Apg16L, a novel WD-repeat protein, targets to the autophagic isolation membrane with the Apg12-Apg5 conjugate. J Cell Sci. 2003;116:1679–1688. 12665549 10.1242/jcs.00381

[39] Kabeya Y, Mizushima N, Ueno T, et al LC3, a mammalian homologue of yeast Apg8p, is localized in autophagosome membranes after processing. Embo J. 2000;19:5720–5728. 11060023 10.1093/emboj/19.21.5720 PMC305793

[40] Tanida I, Ueno T, Kominami E LC3 conjugation system in mammalian autophagy. Int J Biochem Cell Biol. 2004;36:2503–2518. 15325588 10.1016/j.biocel.2004.05.009 PMC7129593

[41] Yamamoto A, Tagawa Y, Yoshimori T, et al Bafilomycin A1 prevents maturation of autophagic vacuoles by inhibiting fusion between autophagosomes and lysosomes in rat hepatoma cell line, H-4-II-E cells. Cell Struct Funct. 1998;23:33–42. 9639028 10.1247/csf.23.33

[42] Cheong H, Yorimitsu T, Reggiori F, et al Atg17 regulates the magnitude of the autophagic response. Mol Biol Cell. 2005;16:3438–3453. 15901835 10.1091/mbc.E04-10-0894 PMC1165424

[43] Noda T, Ohsumi Y Tor, a phosphatidylinositol kinase homologue, controls autophagy in yeast. J Biol Chem. 1998;273:3963–3966. 9461583 10.1074/jbc.273.7.3963

[44] Blommaart EF, Krause U, Schellens JP, et al The phosphatidylinositol 3-kinase inhibitors wortmannin and LY294002 inhibit autophagy in isolated rat hepatocytes. Eur J Biochem. 1997;243:240–246. 9030745 10.1111/j.1432-1033.1997.0240a.x

[45] Klionsky DJ, Abdelmohsen K, Abe A, et al Guidelines for the use and interpretation of assays for monitoring autophagy. Autophagy. 2016;12:1–222. 26799652 10.1080/15548627.2015.1100356 PMC4835977

[46] Bauckman KA, Owusu-Boaitey N, Mysorekar IU Selective autophagy: xenophagy. Methods. 2015;75:120–127. 25497060 10.1016/j.ymeth.2014.12.005 PMC4355331

[47] Pareja MEM, Colombo MI Autophagic clearance of bacterial pathogens: molecular recognition of intracellular microorganisms. Front Cell Infect Microbiol. 2013;3:54. 24137567 10.3389/fcimb.2013.00054 PMC3786225

[48] Gutierrez MG, Munafó DB, Berón W, et al Rab7 is required for the normal progression of the autophagic pathway in mammalian cells. J Cell Sci. 2004;117:2687–2697. 15138286 10.1242/jcs.01114

[49] Nakagawa I, Amano A, Mizushima N, et al Autophagy defends cells against invading group A Streptococcus. Science. 2004;306:1037–1040. 15528445 10.1126/science.1103966

[50] Heckmann BL, Boada-Romero E, Cunha LD, et al LC3-associated phagocytosis and inflammation. J Mol Biol. 2017;429:3561–3576. 28847720 10.1016/j.jmb.2017.08.012 PMC5743439

[51] Lai S, Devenish RJ LC3-associated phagocytosis (LAP): connections with host autophagy. Cells. 2012;1:396–408. 24710482 10.3390/cells1030396 PMC3901117

[52] Veiga-Santos P, Desoti VC, Miranda N, et al The natural compounds piperovatine and piperlonguminine induce autophagic cell death on Trypanosoma cruzi. Acta Trop. 2013;125:349–356. 23228524 10.1016/j.actatropica.2012.11.014

[53] Fernandes MC, Da Silva EN, Pinto AV, et al A novel triazolic naphthofuranquinone induces autophagy in reservosomes and impairment of mitosis in Trypanosoma cruzi. Parasitology. 2012;139:26–36. 21939585 10.1017/S0031182011001612

[54] Dos Anjos DO, Sobral Alves ES, Gonçalves VT, et al Effects of a novel β-lapachone derivative on Trypanosoma cruzi: parasite death involving apoptosis, autophagy and necrosis. Int J Parasitol Drugs Drug Resist. 2016;6:207–219. 27770751 10.1016/j.ijpddr.2016.10.003 PMC5078628

[55] Lazarin-Bidóia D, Desoti VC, Martins SC, et al Dibenzylideneacetones are potent trypanocidal compounds that affect the Trypanosoma cruzi redox system. Antimicrob Agents Chemother. 2016;60:890–903. 26596953 10.1128/AAC.01360-15 PMC4750705

[56] Braga MV, Magaraci F, Lorente SO, et al Effects of inhibitors of Delta24(25)-sterol methyl transferase on the ultrastructure of epimastigotes of Trypanosoma cruzi. Microsc Microanal. 2005;11:506–515. 17481329 10.1017/S143192760505035X

[57] Santa-Rita RM, Lira R, Barbosa HS, et al Anti-proliferative synergy of lysophospholipid analogues and ketoconazole against Trypanosoma cruzi (Kinetoplastida: trypanosomatidae): cellular and ultrastructural analysis. J Antimicrob Chemother. 2005;55:780–784. 15790672 10.1093/jac/dki087

[58] Michels PAM, Bringaud F, Herman M, et al Metabolic functions of glycosomes in trypanosomatids. Biochim Biophys Acta. 2006;1763:1463–1477. 17023066 10.1016/j.bbamcr.2006.08.019

[59] Herman M, Gillies S, Michels PA, et al Autophagy and related processes in trypanosomatids: insights from genomic and bioinformatic analyses. Autophagy. 2006;2:107–118. 16874069 10.4161/auto.2.2.2369

[60] Alvarez VE, Kosec G, Sant’Anna C, et al Autophagy is involved in nutritional stress response and differentiation in Trypanosoma cruzi. J Biol Chem. 2008;283:3454–3464. 18039653 10.1074/jbc.M708474200

[61] Vanrell MC, Losinno AD, Cueto JA, et al The regulation of autophagy differentially affects Trypanosoma cruzi metacyclogenesis. PLoS Negl Trop Dis. 2017;11:e0006049. 29091711 10.1371/journal.pntd.0006049 PMC5683653

[62] Eisenberg T, Knauer H, Schauer A, et al Induction of autophagy by spermidine promotes longevity. Nat Cell Biol. 2009;11:1305–1314. 19801973 10.1038/ncb1975

[63] Schoijet AC, Sternlieb T, Alonso GD The phosphatidylinositol 3-kinase class III complex containing TcVps15 and TcVps34 participates in autophagy in Trypanosoma cruzi. J Eukaryot Microbiol. 2017;64:308–321. 27603757 10.1111/jeu.12367

[64] Li F-J, He CY Acidocalcisome is required for autophagy in Trypanosoma brucei. Autophagy. 2014;10:1978–1988. 25484093 10.4161/auto.36183 PMC4502762

[65] Dorn BR, Dunn WA, Progulske-Fox A Bacterial interactions with the autophagic pathway. Cell Microbiol. 2002;4:1–10. 11856168 10.1046/j.1462-5822.2002.00164.x

[66] Berón W, Gutierrez MG, Rabinovitch M, et al Coxiella burnetii localizes in a Rab7-labeled compartment with autophagic characteristics. Infect Immun. 2002;70:5816–5821. 12228312 10.1128/IAI.70.10.5816-5821.2002 PMC128334

[67] Amer AO, Swanson MS Autophagy is an immediate macrophage response to Legionella pneumophila. Cell Microbiol. 2005;7:765–778. 15888080 10.1111/j.1462-5822.2005.00509.x PMC1584279

[68] Gutierrez MG, Vázquez CL, Munafó DB, et al Autophagy induction favours the generation and maturation of the Coxiella-replicative vacuoles. Cell Microbiol. 2005;7:981–993. 15953030 10.1111/j.1462-5822.2005.00527.x

[69] Romano PS, Gutierrez MG, Berón W, et al The autophagic pathway is actively modulated by phase II Coxiella burnetii to efficiently replicate in the host cell. Cell Microbiol. 2007;9:891–909. 17087732 10.1111/j.1462-5822.2006.00838.x

[70] Vanrell MC, Cueto JA, Barclay JJ, et al Polyamine depletion inhibits the autophagic response modulating Trypanosoma cruzi infectivity. Autophagy. 2013;9:1080–1093. 23697944 10.4161/auto.24709 PMC3722317

[71] Romano PS, Cueto JA, Casassa AF, et al Molecular and cellular mechanisms involved in the Trypanosoma cruzi/host cell interplay. IUBMB Life. 2012;64:387–396. 22454195 10.1002/iub.1019 PMC3709976

[72] Martins RM, Alves RM, Macedo S, et al Starvation and rapamycin differentially regulate host cell lysosome exocytosis and invasion by Trypanosoma cruzi metacyclic forms. Cell Microbiol. 2011;13:943–954. 21501360 10.1111/j.1462-5822.2011.01590.x

[73] Owen KA, Casanova JE Salmonella manipulates autophagy to “serve and protect.” Cell Host Microbe. 2015;18:517–519. 26567504 10.1016/j.chom.2015.10.020

[74] Onizuka Y, Takahashi C, Uematsu A, et al Inhibition of autolysosome formation in host autophagy by Trypanosoma cruzi infection. Acta Trop. 2017;170:57–62. 28232068 10.1016/j.actatropica.2017.02.021

[75] Krieger V, Liebl D, Zhang Y, et al Reorganization of the endosomal system in Salmonella-infected cells: the ultrastructure of Salmonella-induced tubular compartments. PLoS Pathog. 2014;10:e1004374. 25254663 10.1371/journal.ppat.1004374 PMC4177991

[76] López de Armentia MM, Gauron MC, Colombo MI Staphylococcus aureus alpha-toxin induces the formation of dynamic tubules labeled with LC3 within host cells in a Rab7 and Rab1b-dependent manner. Front Cell Infect Microbiol. 2017;7:431. 29046869 10.3389/fcimb.2017.00431 PMC5632962

[77] Castillo EF, Dekonenko A, Arko-Mensah J, et al Autophagy protects against active tuberculosis by suppressing bacterial burden and inflammation. Proc Natl Acad Sci. 2012;109:E3168–E3176. 23093667 10.1073/pnas.1210500109 PMC3503152

[78] Cooper AM, Dalton DK, Stewart TA, et al Disseminated tuberculosis in interferon gamma gene-disrupted mice. J Exp Med. 1993;178:2243–2247. 8245795 10.1084/jem.178.6.2243 PMC2191280

[79] Knop J Immunologic effects of interferon. J Invest Dermatol. 1990;95:72S–74S. 1701812 10.1111/1523-1747.ep12874780

[80] Gutierrez MG, Master SS, Singh SB, et al Autophagy is a defense mechanism inhibiting BCG and mycobacterium tuberculosis survival in infected macrophages. Cell. 2004;119:753–766. 15607973 10.1016/j.cell.2004.11.038

[81] Deretic V, Levine B Autophagy, immunity, and microbial adaptations. Cell Host Microbe. 2009;5:527–549. 19527881 10.1016/j.chom.2009.05.016 PMC2720763

